# Anti-HER2 IgY antibody-functionalized single-walled carbon nanotubes for detection and selective destruction of breast cancer cells

**DOI:** 10.1186/1471-2407-9-351

**Published:** 2009-10-02

**Authors:** Yan Xiao, Xiugong Gao, Oleh Taratula, Stephen Treado, Aaron Urbas, R David Holbrook, Richard E Cavicchi, C Thomas Avedisian, Somenath Mitra, Ronak Savla, Paul D Wagner, Sudhir Srivastava, Huixin He

**Affiliations:** 1 Chemical Science and Technology Laboratory, National Institute of Standards and Technology (NIST), Gaithersburg, MD, USA; 2 Research and Development, Translabion, Clarksburg, MD, USA; 3 Department of Chemistry, Rutgers University, Newark, NJ, USA; 4 Building Environment Division, National Institute of Standards and Technology (NIST), Gaithersburg, MD, USA; 5 Sibley School of Mechanical and Aerospace Engineering, Cornell University, Ithaca, NY, USA; 6 Department of Chemistry and Environmental Science, New Jersey Institute of Technology, Newark, NJ, USA; 7 Division of Cancer Prevention, National Cancer Institute (NCI), Bethesda, MD, USA

## Abstract

**Background:**

Nanocarrier-based antibody targeting is a promising modality in therapeutic and diagnostic oncology. Single-walled carbon nanotubes (SWNTs) exhibit two unique optical properties that can be exploited for these applications, strong Raman signal for cancer cell detection and near-infrared (NIR) absorbance for selective photothermal ablation of tumors. In the present study, we constructed a HER2 IgY-SWNT complex and demonstrated its dual functionality for both detection and selective destruction of cancer cells in an *in vitro *model consisting of HER2-expressing SK-BR-3 cells and HER2-negative MCF-7 cells.

**Methods:**

The complex was constructed by covalently conjugating carboxylated SWNTs with anti-HER2 chicken IgY antibody, which is more specific and sensitive than mammalian IgGs. Raman signals were recorded on Raman spectrometers with a laser excitation at 785 nm. NIR irradiation was performed using a diode laser system, and cells with or without nanotube treatment were irradiated by 808 nm laser at 5 W/cm^2 ^for 2 min. Cell viability was examined by the calcein AM/ethidium homodimer-1 (EthD-1) staining.

**Results:**

Using a Raman optical microscope, we found the Raman signal collected at single-cell level from the complex-treated SK-BR-3 cells was significantly greater than that from various control cells. NIR irradiation selectively destroyed the complex-targeted breast cancer cells without harming receptor-free cells. The cell death was effectuated without the need of internalization of SWNTs by the cancer cells, a finding that has not been reported previously.

**Conclusion:**

We have demonstrated that the HER2 IgY-SWNT complex specifically targeted HER2-expressing SK-BR-3 cells but not receptor-negative MCF-7 cells. The complex can be potentially used for both detection and selective photothermal ablation of receptor-positive breast cancer cells without the need of internalization by the cells. Thus, the unique intrinsic properties of SWNTs combined with high specificity and sensitivity of IgY antibodies can lead to new strategies for cancer detection and therapy.

## Background

Although significant progress has been made in both the understanding and treatment of cancer during the last thirty years, it remains the second leading cause of death in the United States. Non-invasive detection of cancer in its early stages is of great interest since early cancer diagnosis, in combination with precise cancer therapies, could significantly increase the survival rate of patients. Nanomedicine, an emerging research area that integrates nanomaterials and biomedicine, has the potential to provide novel diagnostic tools for detection of primary cancers at their earliest stages, and to provide improved therapeutic protocols. Research in nanomedicine will also lead to the understanding of the intricate interplay of nanomaterials with components of biological systems.

Attaching antibodies or other targeting agents (such as receptor ligands) to the surface of nanocarriers to achieve specific targeting of cancerous cells is a promising modality for therapeutic and diagnostic oncology [1]. Improved therapeutic efficacy of targeted nanocarriers has been established in multiple animal models of cancer, and currently more than 120 clinical trials are underway with various antibody-containing nanocarrier formulations [2]. The most commonly explored nanocarriers include polymer conjugates, polymeric nanoparticles, lipid-based carriers such as liposomes and micelles, and dendrimers [1]. Recent developments in nanotechnology have engendered a range of novel inorganic nanomaterials, such as metal nanoshells [3] and carbon nanotubes [4], offering unique opto-electronic properties compared with conventional organic nanocarriers [3,4].

Single-walled carbon nanotube (SWNT) is a novel nanomaterial that exhibits unique structural, mechanical, electrical and optical properties that are promising for various biological and biomedical applications, such as biosensors [5], novel biomaterials [6], and drug delivery transporters [7-11]. Water-solubilized SWNTs have been shown to transverse the cell membrane via endocytosis to shuttle various cargoes into cells, including proteins [12], nucleic acid such as plasmid DNA [13,14] and short interfering RNA [15], without causing cytotoxicity. Two unique intrinsic properties of SWNTs can be exploited to facilitate cancer detection and therapy. SWNTs have very strong resonant Raman scattering [16] that can be harnessed for cancer cell detection [17-19]. SWNTs absorb NIR light in the 700-1100 nm spectral window to which biological systems are transparent; continuous NIR irradiation of SWNTs attached to cancer cells produces excessive heat in the local environment that can be utilized to achieve selective destruction of these cells without harming normal cells [7,20-22].

To achieve specific targeting of tumor cells for photothermal ablation, SWNTs have been either conjugated to folate to target folate receptors in folate positive cancer cells [7,22] or attached noncovalently (through adsorption) [20] or indirectly via streptavidin-biotin interaction [21] to antibodies targeting specific receptors on cancer cells. Direct covalent attachment of antibodies to SWNTs for specific tumor targeting has also been reported [23], however, using such antibody-SWNT conjugates for specific photothermal ablation of cancer cells with NIR light has not been reported.

All of the antibodies in clinical use today for cancer cell targeting are mammalian IgG monoclonal antibodies [24]. Recently, there has been renewed interest in using avian IgY antibodies as IgG substitutes in immunoassays and clinical applications [25]. IgYs, distinct from IgGs in molecular structure and biochemical features, have many attractive biochemical, immunological and production advantages over IgGs and are suitable for further development [25]. We have recently demonstrated the advantages of using anti-HER2 IgY antibody in detecting breast cancer cells [26]. IgY antibodies provide specific and more sensitive detection of breast cancer cells compared with commercial IgG or IgM antibodies. Coupled with quantum dots, anti-HER2 IgY antibodies have the potential to give quantitative biomarker measurements [26].

In an effort to improve breast cancer detection and therapy, we have developed a novel method which combines the advantages of anti-HER2 IgY antibody with the unique properties of SWNTs. We constructed a HER2 IgY-SWNT complex by directly functionalizing SWNTs with the anti-HER2 IgY antibody through covalent bonding, explored the Raman and NIR optical properties of the complex, and tested its feasibility for detection and selective destruction of cancer cells.

## Methods

### Preparation of the HER2 IgY-SWNT complex

Purified HiPco SWNTs were purchased from Carbon Nanotechnologies (Houston, TX) and solubilized by carboxylation using a microwave-assisted functionalization method described previously [27]. In a typical reaction, ~1 mg of as-received carbon nanotubes were added into 2 ml of a 1:1 mixture of 70% nitric acid and 97% sulfuric acid aqueous solutions in a plastic beaker. The mixture was then subjected to microwave radiation for 2 min. Afterwards, the mixture was diluted with deionized water and centrifuged at 2000 *g *for 15 min to remove insoluble materials. The supernatant was filtered through a Microcon YM-50 centrifugal filter unit (Millipore, Billerica, MA) and rinsed thoroughly with 100 mM MES buffer in order to adjust pH to 4.5. For covalent attachment of HER2 IgY antibody onto SWNTs, 2.0 mg N-(3-Dimethylaminopropyl)-N'-ethylcarbodiimide hydrochloride (EDC), 88.3 mg N-Hydroxysuccinimide (NHS) and 100 μL MES buffer solution (100 mM, pH 4.5) were added to the microwave-functionalized SWNT solution and incubated for 60 min at room temperature. The mixture was then centrifuged in Microcon YM-50 centrifugal filter unit and rinsed with a 100 mM MES buffer solution (pH 6.3) to remove excess EDC, NHS and the byproduct urea. The purified, activated carbon nanotubes on the filter were re-dispersed into a 100 mM MES buffer solution (pH 6.3). Thereafter, 60 μl (1.0 mg/ml) chicken anti-HER2 IgY antibody, prepared as described previously [26], was added into the above solution and reacted for 2 h. Finally, the solution was centrifuged at 25,000 *g *for 20 min to remove the unreacted materials. The collected precipitate was resuspended in PBS buffer (100 mM, pH 7.4) and used for further studies. The concentration of antibody conjugated to SWNTs was determined using BCA protein assay (Pierce, Rockford, IL) following the manufacturer's instructions. The SWNT concentration in the solution was estimated from the absorbance spectrum at 808 nm acquired with a Cary-500 UV-visible-NIR spectrophotometer (Varian, Palo Alto, CA) in double-beam mode.

### Cell culture and treatment

Breast carcinoma cell lines SK-BR-3 and MCF-7 were obtained from ATCC (Manassas, VA) and cultured under conditions as recommended by the supplier. Cells were grown for 24 h to reach ~30-40% confluence, then treated with the HER2 IgY-SWNT complex or SWNT or antibody alone at the final nanotube concentration of 4 mg/L for 24 h under the same culture condition. The cell culture was washed 3 times with fresh medium before NIR irradiation to remove unbound nanotubes, antibodies or the antibody nanotube complex.

### Atomic force microscopy (AFM)

The SWNTs before and after conjugation with anti-HER2 IgY antibody were imaged with a tapping mode Nanoscope IIIa atomic force microscope (Veeco, Chadds Ford, PA). In order to image the SWNTs, 5 μl of the prepared solutions were deposited on freshly cleaved mica. After a 3-5 min incubation, the mica surface was rinsed with 3 drops of deionized water 4 times and dried under a flow of nitrogen. During imaging, a 125 μm long rectangular silicon cantilever/tip assembly was used with a spring constant of 40 N/m, resonance frequency of 315-352 kHz and a tip radius of 5-10 nm. The images were generated by the change in amplitude of the free oscillation of the cantilever as it interacts with the sample.

### Dispersive Raman spectrometric analysis

Raman spectra for the HER2 IgY-SWNT complex solution were collected on a S1000 Raman spectrometer from Renishaw (Hoffman Estates, IL) coupled to a DM LM microscope from Leica (Bannockburn, IL) using a 50× objective. The source was an Ar-ion pumped tunable Ti:sapphire laser from Coherent (Santa Clara, CA) operating at 785 nm. Laser power was 7 mW measured at the sample.

Senterra dispersive Raman spectrometer from Bruker Optics (Billerica, MA) was used to collect Raman spectra from cell cultures. The system consisted of laser excitation at 785 nm focused on the samples through an optical system, producing spectra of Raman shifts, which were evaluated to identify and determine the presence and location of the nanomaterial. Measurements were made using a 10× objective lens, with laser powers ranging from 1 to 25 mW, and exposure times of 10 to 60 s. Various settings were tried in order to determine the optimum signal-to-noise ratio and to avoid damaging the samples from over-heating by the laser.

### NIR irradiation and temperature measurement

A Spectra-Physics diode laser from Newport (Irvine, CA) at a wavelength of 808 nm mounted on a heat sink was employed to heat the samples. The heat sink was controlled by a driver and a temperature controller. Two thermocouples made from 80 μm diameter type K wire from Omega (Stamford, CT) were positioned outside of the beam path to record the temperature response. A program written in LabVIEW (National Instruments, Austin, TX) was used to control the timing and power of the laser and to record the temperature of the two thermocouple junctions. The laser exposure was conducted at a current of 3.0 A in the laser diode which produced an output of 5 W/cm^2^, and the duration was set to 120 s.

**Figure 1:**
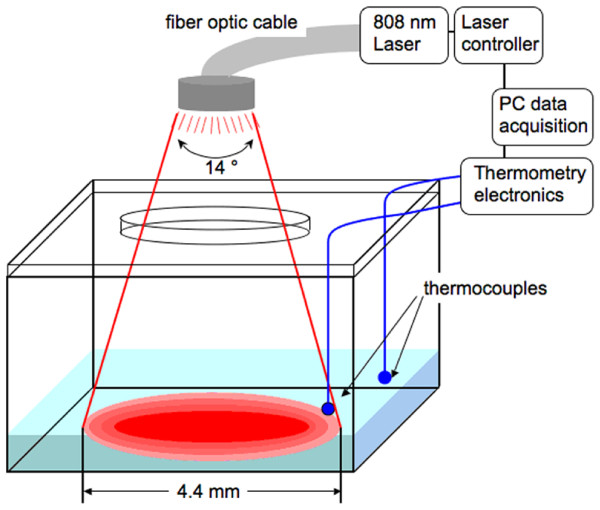
**Schematic of experimental setup for NIR irradiation and temperature measurement**. Dimensions not to scale.

Samples were contained in cells with a 1 cm × 1 cm glass bottom. A removable Teflon cap was fitted over the cells. A 4.5 mm hole was drilled through the cap for the laser to pass through (the beam diameter was 4.4 mm). Two smaller holes were drilled through the cap for the thermocouple wires to pass through. One of the thermocouples was extended below the cap into the fluid and positioned just at the edge of the illuminated area. The second was positioned approximately 1 mm outside the edge of the beam path. The beam passed through the sample's glass bottom and entered a 3 cm diameter beam dump located just below the glass bottom. The beam dump serves to prevent radiation passing through the sample from making its way back to the sample. A diagram for the laser exposure arrangement is shown in Fig. 1.

### Cell viability assay

Ten (10) min after heating with NIR irradiation, cell viability was examined by the calcein AM/ethidium homodimer-1 (EthD-1) staining [28]. The LIVE/DEAD Viability/Cytotoxicity Kit from Molecular Probes (Eugene, OR) was used and protocols provided by the manufacturer were adopted. Cells showing green fluorescence were considered alive; while dead cells showed red fluorescence. Results were expressed as percentage of live cells relative to the number of cells on a control slide that did not go through treatment or NIR irradiation.

### SWNTs localization study by immunohistochemistry (IHC)

Cells were grown on tissue culture chamber slides (Nunc, Rochester, NY) at a density of 30,000 cells/cm^2 ^and then treated with the HER2 IgY-SWNT complex at the final nanotube concentration of 4 mg/L for 24 h. Cell monolayers were subsequently fixed in 10% neutral-buffered zinc formalin (Fisher, Pittsburgh, PA), and were pre-blocked with 5% (w/v) nonfat dry milk in TBST (50 mM Tris-HCl, 150 mM NaCl, 150 mM Tween 20), 20°C, for 20 min. For detection, slides were robotically prepared (reaction with secondary antibody and fluorescent detection reagents) with a Benchmark XT workstation (Ventana, Tucson, AZ) [29]. Anti-IgY biotinylated antibody (GenWay, San Diego, CA) was used as the secondary antibody and was detected by fluorescence microscopy with streptavidin-Qdot655 (Invitrogen, Carlsbad, CA). Imaging systems for analysis of fluorescence signals from quantum dots and integration of the signal with an imaging system were described elsewhere [30,31].

Confocal laser scanning microscopy images were obtained on a TCS SP5/DM6000 from Leica using an HCX Pl Apo oil immersion 63× coverslip corrected objective. A 405 nm Diode laser was used as the excitation source while the emission bands were set to 440 nm to 480 nm (DAPI, channel 1), 640 nm to 660 nm (QDs, channel 2), and diffraction (cells, channel 3). Zoom functions between 1× and 6× were used as needed.

### Data analysis

All experiments were repeated at least 3 times with at least 3 replicates each time. For comparative studies, one-way ANOVA tests (with Bonferroni post test if *p *< 0.05) were used for statistical analysis. Differences were considered statistically significant if a *p *value of < 0.05 was achieved.

## Results

### Preparation and characterization of the HER2 IgY-SWNT Complex

**Figure 2:**
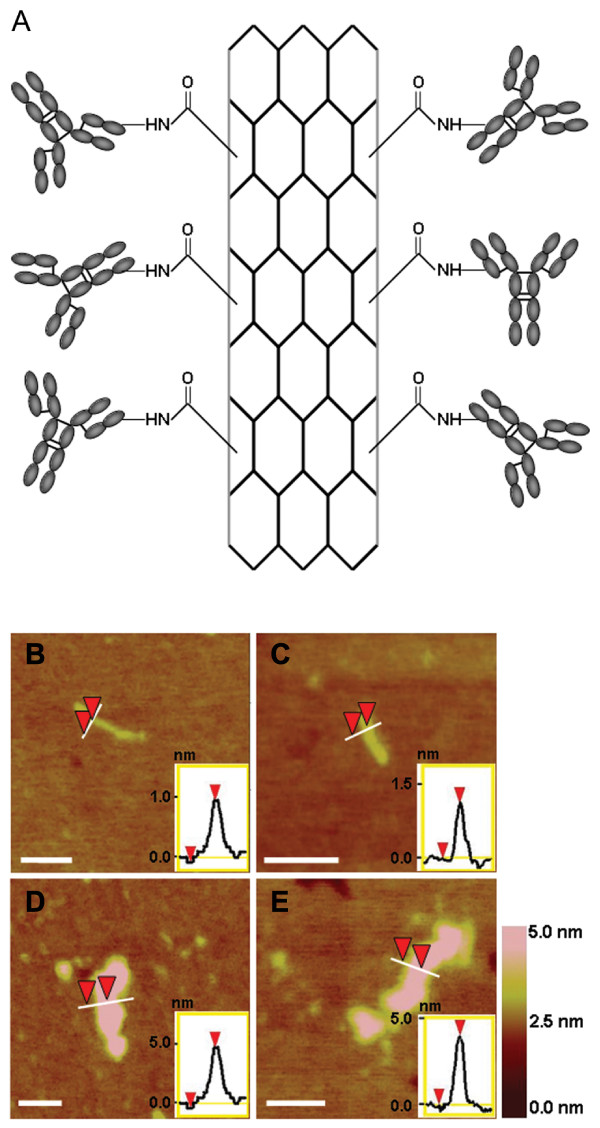
**The HER2 IgY-SWNT complex**. (A) Schematic representation of SWNTs covalently functionalized with anti-HER2 IgY antibody. (B-E) atomic force microscopy (AFM) images of carboxylated SWNTs prior to conjugation (B and C) and after conjugation (D and E) to anti-HER2 IgY antibodies. Insets shows AFM cross-section analysis indicating the changes in height of SWNTs prior to and after conjugation with anti-HER2 IgY antibodies. The height differences on the surface are indicated by the color code shown on the right. Scale bars represent 60 nm.

The HER2 IgY-SWNT complex was prepared by first carboxylating HiPco SWNTs using a microwave-assisted functionalization method published previously [27]; the carboxylated SWNTs were then activated by N-(3-Dimethylaminopropyl)-N'-ethylcarbodiimide hydrochloride (EDC) and *N*-Hydroxysuccinimide (NHS) and reacted with HER2 IgY antibody to form the covalent complex, through amidation between the carboxyl groups on the SWNTs with primary amines on amino acid residues such as lysine and arginine on the antibody (Fig. 2A) [32]. Free unconjugated antibodies were removed through ultracentrifugation. The SWNTs used consisted of short, straight fragments (with average diameter and length being 1.17 ± 0.28 nm and 88.00 ± 43.68 nm, respectively) and exist as individual tubes and small bundles rather than large aggregates as evidenced by atomic force microscopy (AFM) image (Figs. 2B and 2C). After antibody attachment, the diameter of the nanotubes increased to 4.02 ± 0.82 nm (Figs. 2D and 2E). Based on the concentrations of the carbon nanotubes and the IgY antibodies used, it was estimated that on average about 10 IgY antibody molecules were attached to each nanotube. The nanotube complex solutions were highly stable in PBS buffer, without forming aggregates for several months when kept at 4°C.

**Figure 3:**
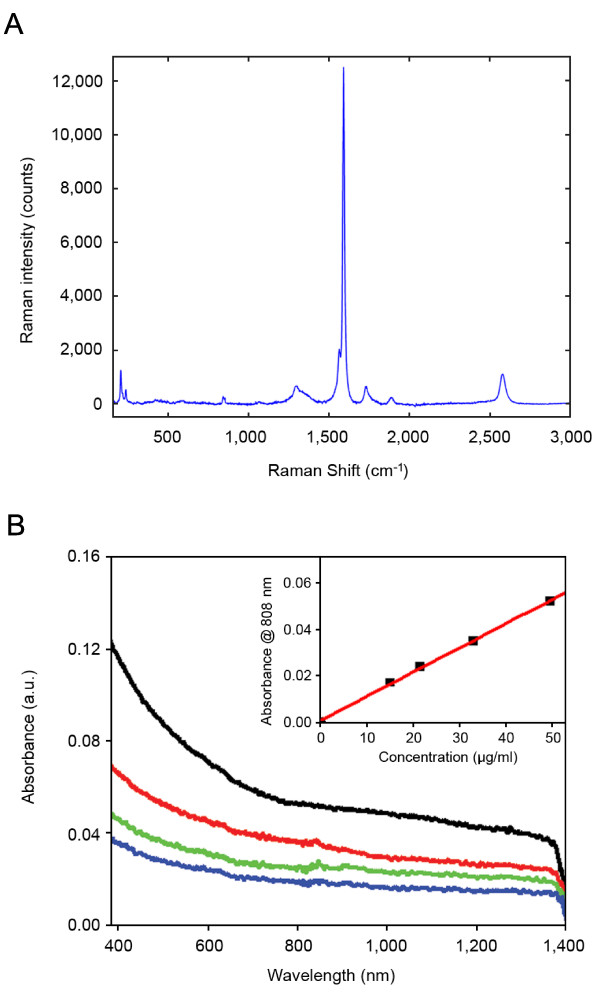
**Optical properties of the HER2 IgY-SWNT complex**. (A) Raman spectra. (B) UV-visible-NIR spectra at different nanotube concentrations (from top to bottom: 49.70, 33.02, 21.61 and 15.11 μg/ml). Inset shows the linear relationship of the absorption at 808 nm versus concentration (optical path = 0.3 cm).

The optical properties of the freshly prepared HER2 IgY-SWNT complex were tested. The Raman spectra (Fig. 3A) of the complex showed a number of well characterized resonances such as the radial breathing mode (RBM) region between 100 and 300 cm^-1 ^and the tangential (G-band) peak at 1,590 cm^-1^. A narrow G^- ^feature was also visible in the G-band region, confirming the presence of semiconducting SWNTs in the sample. The spectra also contained the disorder-induced D band around 1300 cm^-1^. The UV-visible-NIR spectra (Fig. 3B) indicated that the HER2 IgY-SWNT complex has fairly strong absorbance in the NIR region (700-1100 nm spectral window), even though the interband absorption peaks, originating from electronic transitions between the first and second van Hove singularities of the nanotubes [33,34] were smeared out during the microwave dispersing and IgY functionalization process. Thus, SWNTs covalently functionalized with antibody retained a significant portion of their optic properties that are potentially useful for biomedical applications.

### Raman spectrometric detection of cancer cells using the HER2 IgY-SWNT complex

**Figure 4:**
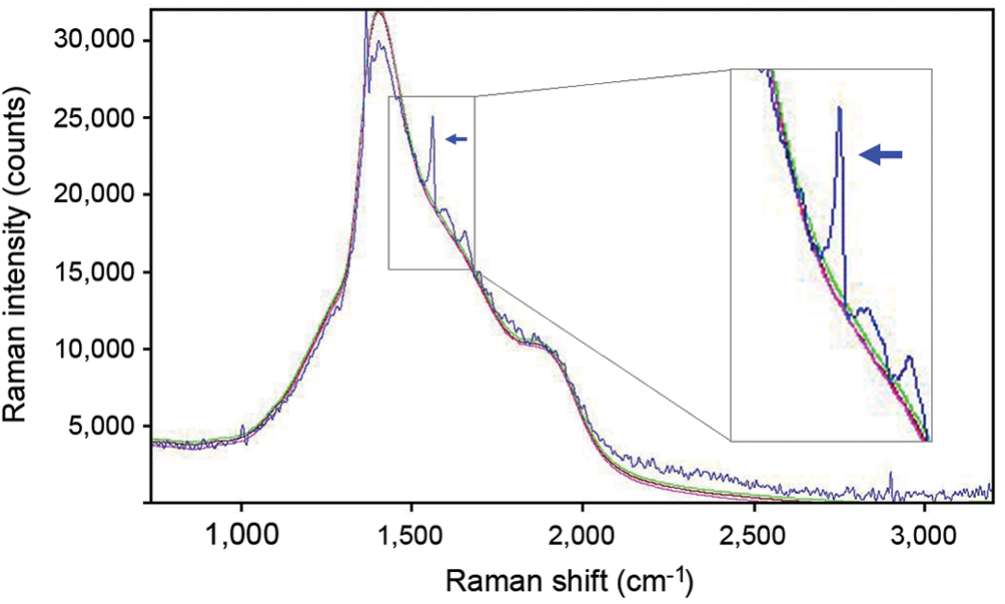
**Raman spectra of breast cancer cells treated by the HER2 IgY-SWNT complex**. A 785 nm laser diode was used for excitation at 1-25 mW through a 10× objective lens on randomly selected cells. Raman spectra of a representative cell from each sample are shown. Blue line, SK-BR-3 cell treated with the HER2 IgY-SWNT complex; red line, SK-BR-3 cell treated with SWNT alone; magenta line, untreated SK-BR-3 cell; green line, MCF-7 cell treated with the HER2 IgY-SWNT complex. The Raman signal indicated by the arrow is the characteristic G band at ~1590 cm^-1^. Inset shows higher resolution spectrum in the area around 1590 cm^-1^.

We first explored the feasibility of harnessing the characteristic ~1590 cm^-1 ^Raman band for *in vitro *specific detection of cancer cells. Breast carcinoma SK-BR-3 cells, which have high HER2 expression [26], were treated with the HER2 IgY-SWNT complex for 24 h. Raman spectroscopy collected at single-cell level from randomly selected cells showed the characteristic G band at ~1590 cm^-1 ^(Fig. 4). The Raman signal from the complex-treated breast cancer cells resulted from the specific binding of the IgY antibody moiety of the complex to the HER2 receptor on the cancer cells, as the same cells treated with SWNTs alone did not exhibit Raman scattering. In addition, MCF-7, which are negative for HER2 expression [26], did not exhibit Raman signals when treated with the HER2 IgY-SWNT complex. Thus the characteristic Raman band at ~1590 cm^-1 ^from the HER2 IgY-SWNT complex differentiated HER2-expressing SK-BR-3 cells from the receptor-negative MCF-7 cells.

### NIR irradiation-induced heating of the HER2 IgY-SWNT complex suspension

**Figure 5:**
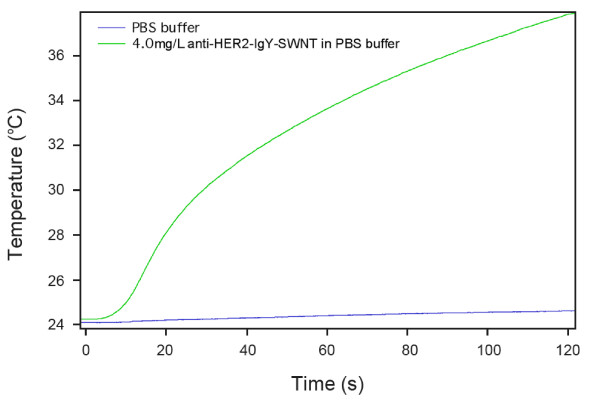
**Temperature measurement during NIR irradiation**. The green line records the temperature evolution of the HER2 IgY-SWNT complex solution at a concentration of 4 mg/L during continuous irradiation by a 808-nm laser at 5.0 W/cm^2 ^for 2 min. The blue line records that for the phosphate-buffered saline (PBS) solution.

To demonstrate the heating effect of the HER2 IgY-SWNT complex upon NIR irradiation, we carried out a control experiment in which an aqueous solution of the HER2 IgY-SWNT complex in PBS at a concentration of 4.0 mg/L was irradiated for 2 min using a laser diode with a wavelength of 808 nm at 5.0 W/cm^2 ^(Fig. 5). The temperature rose rapidly after a short lag of a few seconds then increased constantly with time. The maximum temperature increase was ~14°C. On the other hand, PBS solution without SWNTs showed very little temperature rise (<1°C), indicating the solution is transparent to the 808 nm NIR light.

### Selective photothermal ablation of cancer cells using the HER2 IgY-SWNT complex

**Figure 6:**
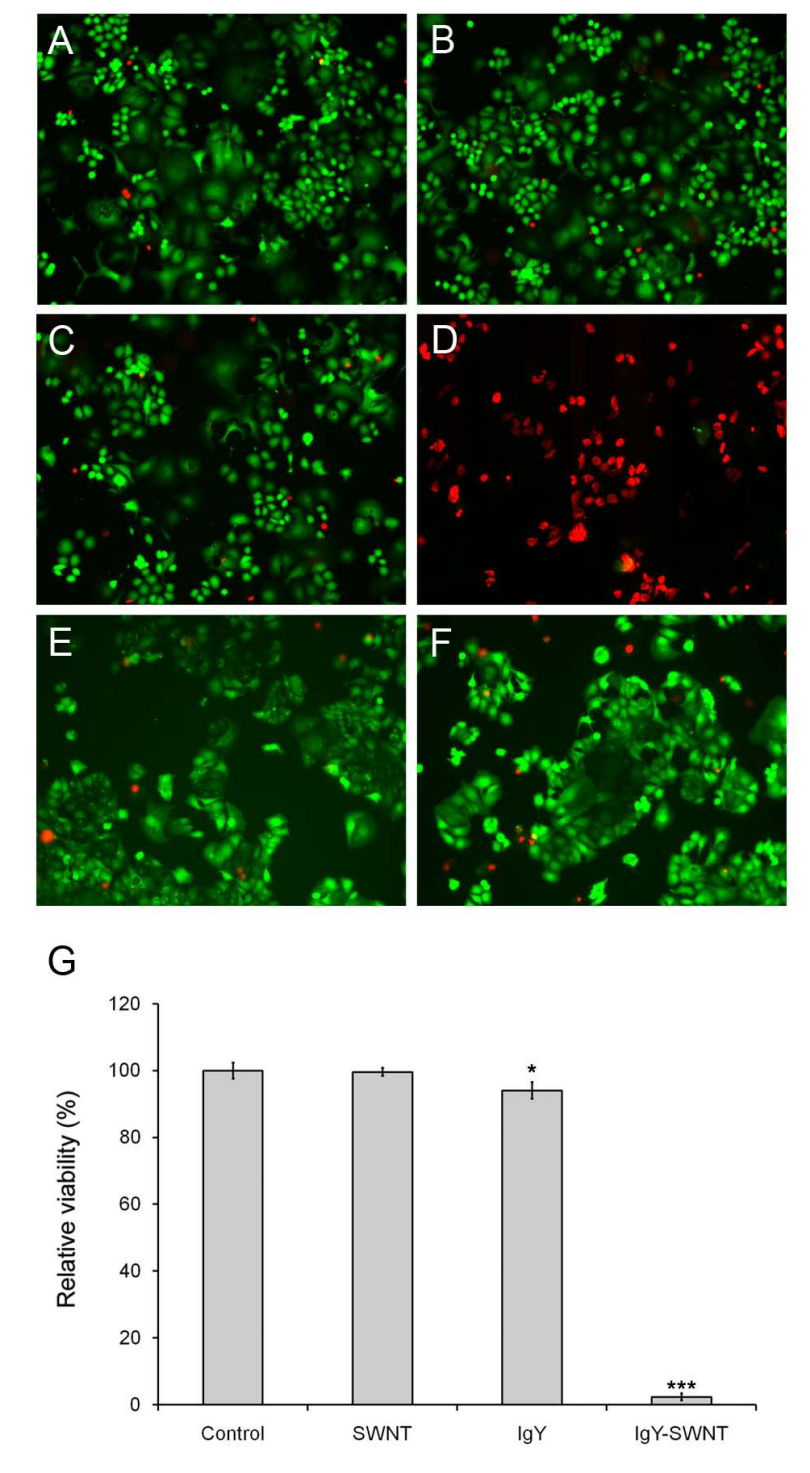
**Cell viability after treatment with the HER2 IgY-SWNT complex followed by NIR irradiation**. Cell viability was examined by calcein AM/EthD-1 fluorescence staining and representative images from each sample are shown. Cells with green fluorescence were considered alive, whereas those with red fluorescence were dead. (A) Untreated SK-BR-3 cells. (B) SK-BR-3 cells treated with SWNT alone. (C) SK-BR-3 cells treated with anti-HER2 IgY antibody alone. (D) SK-BR-3 cells treated with the HER2 IgY-SWNT complex. (E) Untreated MCF-7 cells. (F) MCF-7 cells treated with the HER2 IgY-SWNT complex. All treatments were for 24 h, followed by NIR irradiation with a 808 nm laser at 5 W/cm^2 ^for 2 min. Magnification for all the images (A-F) was 10×. (G) Bar graph showing the percentage of live cells in each sample of SK-BR-3 cells following NIR irradiation. Cells were counted under microscope for 3 randomly selected view fields, and the total number was used for the calculation. The experiment was repeated for 3 times (*n *= 3). Error bars represent standard deviation. Cells that did not go through treatment and irradiation were used as controls (852 ± 20, 100%). * *p *< 0.05 and *** *p *< 0.001 versus control.

Next, we explored the feasibility of using the HER2 IgY-SWNT complex for *in vitro *selective destruction of breast carcinoma SK-BR-3 cells (Fig. 6). We conducted the NIR irradiation with a 808 nm laser at 5 W/cm^2 ^for 2 min. SK-BR-3 cells treated with the HER2 IgY-SWNT complex showed extensive cell death after heating with NIR irradiation (Figs. 6D and 6G); in stark contrast, negligible cell death was observed with SK-BR-3 cells treated with SWNTs alone (Figs. 6B and 6G) or untreated (Figs. 6A and 6G), and in MCF-7 cells treated with the HER2 IgY-SWNT complex (Fig. 6F). These results clearly demonstrated the high transparency of biosystems to NIR light in the vicinity of 808 nm, and at the same time indicated that the specific binding of the IgY antibody moiety of the complex with HER2 receptors on the SK-BR-3 cells is essential for the selective thermal ablation of tumor cells. On the other hand, the SWNT moiety is equally indispensable for the hyperthermia effect, as cell death observed in SK-BR-3 cells treated with the IgY antibody alone (5.9%; Figs. 6C and 6G), although statistically significant (*p *= 0.040), was to a much less extent than in cells treated with the complex (97.7%, *p *= 3.38 × 10^-7^; Figs. 6D and 6G).

### Localization of the HER2 IgY-SWNT complex on the cell membrane

**Figure 7:**
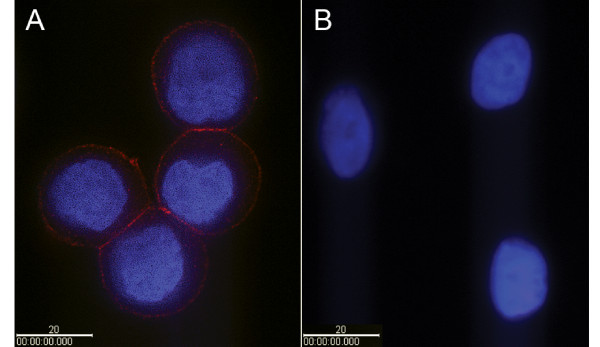
**Localization of the HER2 IgY-SWNT complex on the cell membrane**. (A) SK-BR-3 cells. (B) MCF-7 cells. The HER2 IgY antibody on the complex was probed by biotinylated anti-IgY antibody and detected by fluorescence microscopy with streptavidin-Qdot655 fluorophores. Bars represent 20 μm.

To localize the HER2 IgY-SWNT complexes in the cancer cells, we first performed an immunohistochemical experiment using quantum dots as detection agent. As shown in Fig. 7A, most of the HER2 IgY-SWNT complexes were localized on the membrane of the SK-BR-3 cells forming a shell-like shape, with little detected inside the cells. Fluorescence signal computed from 10 randomly selected cells shows that the intensity ratio of fluorescence on the cell surface to that inside the cell is (563 ± 35): 1. No fluorescence signal was detected in receptor-free MCF-7 cells (Fig. 7B), suggesting that binding of the complex onto SK-BR-3 cells resulted from the anti-HER2 activity of its antibody moiety.

**Figure 8:**
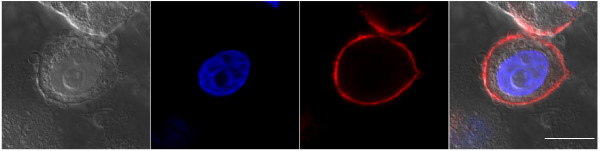
**Confocal microscopic visualization confirming the localization of the HER2 IgY-SWNT complex on the cell membrane of SK-BR-3 cells**. From left to right, the panels are the brightfield image, DAPI channel image showing the nucleus, quantum dot (QD) channel image showing fluorescence from the HER2 IgY-SWNT complex, and an overlay of the three. The white bar represents 10 μm.

To confirm the above result, we performed additional experiments using confocal microscopy for imaging. The high-resolution images shown in Fig. 8 clearly demonstrated that the HER2 IgY-SWNT complexes were localized on the membrane of the SK-BR-3 cells and were not internalized by the cancer cells.

## Discussion

The first problem to tackle for biomedical applications of SWNTs is to solubilize and disperse carbon nanotubes in aqueous solutions and functionalize them with biomolecules such as proteins/antibodies, nucleic acids and carbohydrates. Past studies using SWNT-antibody conjugates for specific photothermal ablation of cancer cells attached antibodies to SWNTs either noncovalently through adsorption [20] or indirectly via streptavidin-biotin interaction [21]. Direct adsorption of antibodies to SWNTs is simple to execute but the weak interaction between the antibody and the nanotubes raises the possibility of loss of the targeting function of the antibodies. Indirect conjugation via streptavidin-biotin interaction involves an additional step of preparing the antibody-biotin complex. A method for direct covalent attachment of antibodies to SWNTs for specific tumor targeting has been reported [23] that involves four reactive steps. Here, we used a simpler method for direct covalent conjugation of antibody to SWNTs. HiPco SWNTs were first dispersed in water through microwave-assisted carboxylation, activated by EDC and NHS, and reacted with HER2 IgY antibody to form the covalent complex. Microwave-assisted functionalization has several advantages over conventional chemical techniques, such as rapidness and environmental friendliness [27]. However, the functionalization process causes some changes in the optoelectronic properties of the SWNTs, such as increase in the disorder mode (D-band) at ~1300 cm^-1 ^(Fig. 3A) and loss of interband transitions between van Hove singularities in the absorption spectrum (Fig. 3B). Similar changes have been reported previously for covalently functionalized carbon nanotubes [27,35,36]. Nevertheless, the resultant IgY-SWNT complexes retain a significant portion of the optic properties of SWNTs, as evidenced by the fairly strong Raman and NIR absorbance.

The characteristic G band at ~1590 cm^-1 ^was detected in HER2-expressing SK-BR-3 cells treated with the IgY-SWNT complexes (Fig. 4) but not in the similarly treated receptor-negative MCF-7 cells, indicating the ability of Raman spectroscopy to specifically detect cancer cells *in vitro*. As a nondestructive optical spectroscopic technique that does not require extrinsic contrast-enhancing agents, the use of Raman spectroscopy has seen a remarkable increase during the last decade in its application to the field of medicine [37]. In particular, Raman spectroscopy has shown great promise as a new tool for detection of malignant and premalignant tissues and as a real-time guidance tool during oncosurgical procedures [38]. However, most of these studies are based on spectral differences between normal and neoplastic tissues that result from compositional changes in the affected tissues, and thus, in most cases, the detection is not highly specific and only possible at later stages of tumor progression. In the current study, characteristic Raman signals (at ~1590 cm^-1^) are collected at the single-cell level from cancer cells targeted by the IgY-SWNT complexes, thus opening the possibility of using Raman spectroscopy for targeted molecular detection of tumors at the incipient stage. An added advantage of Raman spectroscopy lies in its potential for *in vivo *applications for which limited penetration depth is a fundamental barrier. Until recently, Raman spectroscopy has been generally restricted to probing surface or near-surface areas of biological tissues with penetration depth of only several hundred microns into tissue. This limitation mainly stems from the diffuse scattering nature of tissue which leads to random propagation of photons within its matrix and prevents the formation of sharp images required to discriminate signals emerging from deeper areas. Several methods have been developed recently for the retrieval of Raman signals from deep areas thus enhancing tissue penetration of Raman spectroscopy. These deep Raman techniques discriminate between Raman signals emerging from different depths within the sample using temporal or spatial gating [39]. For instance, combining spatially offset Raman spectroscopy (SORS) with three-dimensional tomographic imaging, it was possible to image a canine hind limb section of a thickness of up to 45 mm using transmission Raman spectroscopy [40,41]. Therefore, combined with advances in Raman spectroscopic technologies for deep tissue imaging [39], SWNTs functionalized with antibody specific for tumor cell receptors may be exploited for *in vivo *specific detection of cancer cells at early stages.

The present study demonstrates very high specificity of the HER2 IgY-SWNT complexes for HER2-expressing cancer cells, indicating the potential usefulness of the IgY antibody for selective targeting of cancer cells. IgY antibodies offer many advantages over their mammalian IgG counterparts in terms of both production and biochemical and immunological properties. IgY antibodies can be isolated in large quantities from egg yolk using simple separation methods; the non-invasive production method also brings the great benefit concerning the welfare of the immunized animals [42]. IgY antibodies can also be used to avoid interference in immunological assays caused by the human complement system, rheumatoid factors, human anti-mouse IgG antibodies (HAMA) or human and bacterial Fc-receptors [43]. Similarly, for clinical use as antibody-based therapeutics, they neither activate mammalian complement nor interact with mammalian Fc receptors that could mediate inflammatory responses [44]. Despite these advantages, the application of IgY antibodies in research and medicine has been very limited [45]. Oral administration of IgY antibodies have shown great promise as immunotherapy for the prevention and treatment of enteric, respiratory, and dental infections in humans and animals [44-47]. As eggs are normal dietary components, there is practically no risk of toxic side effects of oral administration of IgY antibodies [44,46]. However, the phylogenetic distance between birds and mammals implies potential concerns over the immunogenicity of IgY antibodies in human. So far, there has been no report on intravenous administration of IgY antibodies in human and the associated immune responses. Nevertheless, concerns over IgY immunogenicity in human should be completely cleared out before any clinical application of IgY should be attempted. The results presented here and in a previous study [26] may bring more attention to this class of antibodies and promote studies on the immunogenicity of IgY preparations in human.

Temperature measurement of the IgY-SWNT complex solution at the nanotube concentration of 4 mg/L showed an increase of ~14°C in the bulk solution, indicating the temperature rise of the surrounding environment would not cause harm to normal cells that do not bind to the SWNT-containing complex in the short time period (2 min). On the other hand, the same result also hinted that the thermal destructive effect to cancer cells must be microscopic rather than macroscopic. We hypothesize that temperature rise in the nanoscale vicinity of individual nanotubes can be dramatic. The sharp local temperature increase may cause damage to subcellular structures such as cell membranes ultimately leading to cell death. The ability to directly measure temperature of an individual nanoparticle will help to validate the hypothesis, and such an endeavor is currently underway [48].

The method described here for selective cancer cell destruction differs from the previously published ones [7,20-22] in that our method does not require internalization of SWNTs into tumor cells. HER2 is a transmembrane glycoprotein with the receptor motif extended outside the cell membrane [49]. The reason for the lack of internalization of the SWNT complex by the cancer cells after binding to the cell surface receptor is not known; however, it is likely due to the surface chemistry of the SWNTs used here [27,32]. It has been reported the surface chemistry has a profound impact on the cellular uptake of nanoparticles such as quantum dots [50]. Although the exact mechanism may differ for various nanoparticles, the surface dependent cellular uptake may be a common phenomenon for all nanoparticles [51]. It is very important to note that the functionalization method used in the current study is different from those published previously where internalization of SWNTs after binding to the cell surface receptors have been reported [7,20,22]. In the study by Chakravarty *et al*. [21], cellular localization of SWNTs after incubation with cancer cells was not reported.

The method described here for selective photothermal ablation of cancer cells without the need of internalization by the cells has the advantage of being more easily extended to other types of cancer cells over agents that need internalization, as cellular internalization is not always achievable with all cancer types. Many cancer cells overexpress specific tumor markers (receptors) on their surface for which IgY antibody with high specificity and sensitivity can be developed. Thus, the IgY-SWNT complex, as exemplified in this study by the anti-HER2 IgY antibody, has the potential to become a novel, generic modality for detection and therapy of various cancer types. Our next step is to evaluate the pharmacokinetics, biodistribution, cytotoxicity and activity of such IgY-SWNT complexes *in vivo *using animal models.

## Conclusion

Our current work exploited two unique optical properties of SWNTs - very strong Raman signals and very strong NIR absorbance. We constructed a HER2 IgY-SWNT complex by covalently functionalizing SWNTs with anti-HER2 IgY antibody to impart to SWNTs the high specificity and sensitivity of the IgY antibody. The resultant complex was successfully used *in vitro *for both detection and selective destruction of HER2-expressing breast cancer cells. Raman signal from cancer cells was detected at the single-cell level. A uniqueness of this dual-function agent is that it does not require internalization by the cancer cells in order to achieve the selective photothermal ablation, thus offering the advantage of being more easily extended to other types of cancer cells. However, further research is needed before these findings can be translated into clinical trials.

## Competing interests

The authors declare that they have no competing interests.

## Authors' contributions

YX conceived of the study, designed and carried out most of the experimental work, coordinated the project, analyzed the data, and drafted the manuscript. XG participated in the design of the study, in the IgY antibody design and production, performed data analysis, and drafted the manuscript. OT carried out HER2 IgY-SWNT complex preparation and characterization, and analyzed the data. ST and AU participated in the Raman spectrometry studies. RDH participated in confocal imaging studies. REC and CTA participated in NIR irradiation and temperature measurement studies. SM prepared the SWNT samples. RS participated in HER2 IgY-SWNT complex preparation. PDW and SS participated in the design of the study and critically revised the manuscript. HH participated in the design of the study, supervised the HER2 IgY-SWNT complex preparation and characterization, and helped to draft the manuscript. All authors read and approved the final manuscript.

## Pre-publication history

The pre-publication history for this paper can be accessed here:

http://www.biomedcentral.com/1471-2407/9/351/prepub
